# Trends, challenges, and outcomes of extrapulmonary tuberculosis: a ten-year study in Cologne (2012–2022)

**DOI:** 10.1186/s12889-025-25835-x

**Published:** 2025-12-12

**Authors:** Dominic Rauschning, Jomana Reusch, Natalie Funke, Clara Lehmann, Eva Scharnowski, Angela Klingmüller, Alexander Simonis, Victor Suárez, Julia Fischer, Gerd Fätkenheuer, Margot Denfeld, Martin Hellmich, Jan Rybniker, Florian Neuhann, Isabelle Suárez

**Affiliations:** 1 Department I of Internal Medicine, Division of Infectious Diseases, University of Cologne, Kerpener Str. 6, Cologne, 50937 Germany; 2 Department IB of Internal Medicine, Division of Infectious Diseases, Bundeswehr Central Hospital Koblenz, Koblenz, Germany; 3 Institute of Medical Statistics and Computational Biology, Faculty of Medicine, University Hospital Cologne, University of Cologne, Cologne, Germany; 4 Health department of the City of Cologne, Cologne, Germany; 5 German Center for Infection Research (DZIF), Partner Site Bonn-Cologne, Cologne, Germany; 6 Center for Molecular Medicine Cologne (CMMC), Medical Faculty and University Hospital Cologne, University of Cologne, Cologne, Germany; 7 MVZ Hautarztpraxis Wilmersdorf, Berlin, Germany; 8 Department II of Internal Medicine (Nephrology, Rheumatology, Diabetes, and General Internal Medicine), University of Cologne, Cologne, Germany; 9 Emergency Department, Faculty of Medicine, University of Cologne, Cologne, Germany; 10 Department for Internal Medicine B, University Hospital Münster, Münster, Germany; 11 Department of Medical Statistics, University Medical Center Gööttingen, Gööttingen, Germany; 12 Faculty of Medicine and University Hospital, Heidelberg Institute of Global Health (HIGH), Heidelberg University, Heidelberg, Germany; 13 School of Medicine and Clinical Sciences Levy Mwanawasa Medical University, Lusaka, Zambia

**Keywords:** Extrapulmonary tuberculosis, Incidence, Diagnostics, Treatment, Migration

## Abstract

**Background:**

Extrapulmonary tuberculosis (EPTB) presents significant challenges in diagnosis and treatment and generally receives less attention than pulmonary tuberculosis (PTB).

**Methods:**

We conducted a retrospective analysis of tuberculosis (TB) patients reported to the public health department of Cologne from 2012 to 2022 focussing on EPTB, its epidemiology, diagnostic methods, and treatment protocols, within a major German city. A subgroup analysis of EPTB patients (2012–2019, *n* = 254) examined diagnostic accuracy, treatment regimens, and adherence.

**Results:**

Of 1,003 notified TB diagnoses, 33% (329/1,003) were identified as EPTB, with lymph nodes being the most frequently affected site. *Mycobacterium tuberculosis* was the predominant pathogen. EPTB was significantly more prevalent among individuals from countries with high TB incidence rates (41%, *p* < 0.001) compared to those from regions with moderate or low incidence. Direct pathogen detection was most frequently achieved by tissue culture (163/199, 82%), followed by polymerase chain reaction (PCR; 161/205, 79%). The standard combination therapy was used in > 95% of patients, aligning to national guidelines.

In contrast to declining PTB incidence, EPTB remained stable. Overall documented treatment success rate (79%) fell short of the WHO target (≥90%), underscoring the need for improved case management and reporting strategies.

**Conclusions:**

Enhanced awareness, specialised care, and targeted interventions for migrant populations are critical to achieving global TB control objectives.

## Introduction

The globally endorsed “End TB” strategy aims to reduce tuberculosis (TB) incidence and mortality through enhanced national surveillance systems, improved diagnostics, and strengthened treatment programs [1]. In Western Europe, incidence and mortality of TB have declined over the years [2]. Despite this trend, emerging factors such as the HIV pandemic, international migration and multidrug resistant strains of mycobacteria have refocused attention on this persistent threat [3]. In Germany, TB is a notifiable disease and the Robert Koch Institute (RKI) oversees nationwide TB surveillance, capturing over 90% of estimated cases [4].

While in Germany extrapulmonary TB (EPTB) accounts for 23.9% [5], EPTB can comprise over 40% of regional TB cases in Middle Eastern countries [6]. Migration is recognised as a contributing factor in the prevalence of EPTB in low-incidence countries [7, 8].

As the fourth-largest city in Germany, Cologne has historically functioned as a central node for migration flows [9]. This study aims to examine the epidemiology of EPTB in Cologne over a 10-year period, focusing on incidence trends, diagnostic challenges, and vulnerable demographic groups. To achieve this, we supplemented mandatory reporting data with detailed clinical information on EPTB patients.

## Methods

### Study population and data acquisition

**Fig. 1:**
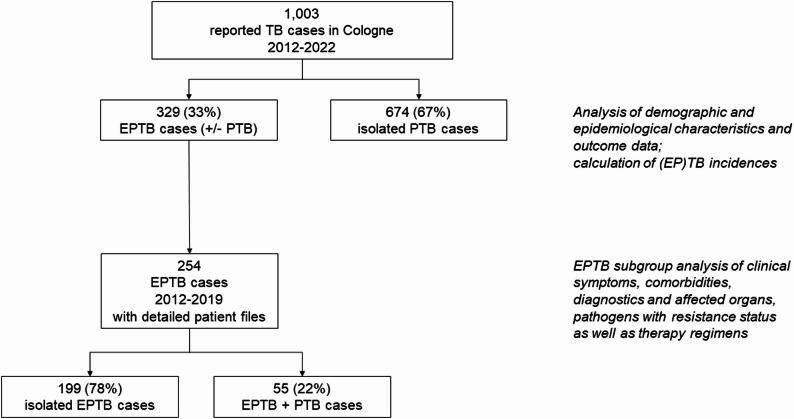
Study outline with numbers and percentages of included cases. TB, tuberculosis; EPTB, extrapulmonary tuberculosis; PTB, pulmonary tuberculosis

The study was conducted through a collaboration between the University Hospital of Cologne and the Municipal Health Department Cologne. We retrospectively analysed the notified TB cases reported by the health authority between January 1 st 2012 and December 31 st 2022 as shown in Fig. 1.

Data were extracted from the municipal reporting software Octoware^®^TN (easy-soft GmbH Dresden, Germany), encompassing epidemiological information such as date of birth, gender, nationality, place of birth, and residence status, along with disease-specific details, including the date of diagnosis, site of TB manifestation, resistance profiles, and treatment outcomes.

The residence status “asylum seeker” was documented starting from 2016 based on an internal directive. However, from 2012 to 2015, it remains uncertain whether all asylum seekers were consistently recorded as such.

The Cologne health authority had acquired additional clinical data on EPTB cases until the end of 2019, when the SARS-CoV-2 pandemic began to consume administrative capacities. In addition to the reported data, these files comprised discharge reports, microbiological findings and progress reports from treating physicians. Consequently, additional insights into EPTB, including comorbidities, treatment regimens and modifications, as well as treatment-related side effects were obtained and used for subgroup analysis (Fig. 1).

### Statistical analysis

Study characteristics are reported as absolute numbers and percentages, medians plus interquartile range (IQR) or means plus/minus standard deviation (SD), as appropriate. Statistical significance was considered at *p*-values < 0.05, demanding 95% confidence intervals (95% CI). Categorical variables were described using frequencies and percentages; comparison was carried out by χ²-test for independence or Fisher’s exact test. Continuous variables were analysed using Mann-Whitney-U-Test or t-test as appropriate. All analysis were performed by IBM SPSS Statistics (Version 29.0.2.0, IBM Corporation, New York, USA).

The regions of origin were categorised into WHO regions or countries with an estimated TB incidence of < 10, 10–100, and >100 per 100,000 population, according to the WHO Global TB Report concerning the year of TB diagnosis. Local incidences were calculated by using the publicly available population data from the Office for Urban Development and Statistics of the City of Cologne [10, 11].

Detected drug resistance was categorised according to international categories “mono-resistance”, “poly-resistance”, “multi drug resistance (MDR)” and “(pre) extensive drug resistance (XDR)” related to the standard therapy consisting of isoniazid (INH), rifampicin (RMP), pyrazinamide (PZA) and ethambutol (EMB) [12, 13].

We categorised treatment outcomes consistent with those reported and employed by the RKI: Successful treatment included cure or at least complete therapy. A distinction was made between continuing, discontinued and failed treatment as well as between death from TB and other causes [5]. Patients who relocated to another jurisdiction during treatment, leading to an unknown final treatment outcome, were categorised separately.

To investigate the impacts of the 2015 refugee crisis and the COVID-19 pandemic, Interrupted Time Series Analysis (ITSA) was applied, based on the multiple step approach of James Lopez Bernal et al. [14]. The development of the data collected after an event is compared with the assumed development calculated from the data collected before the event. The method then evaluates changes in slope and level of the time series; significant differences indicate effects of the event. Given that 2012 marked the year with the lowest TB incidence ever reported in Germany and considering the relatively short time span between the study commencement and the event, we augmented the analysis by incorporating regional incidences reported by the RKI for the city of Cologne from 2008 to 2011 [15, 16] for the ITSA of the 2015 migration crisis.

## Results

### Cohort overview: demographics, epidemiology, drug resistance patterns, and outcomes

Out of 1,003 notified and confirmed TB, EPTB accounted for 26% (261/1,003) and extrapulmonary manifestations with concomitant PTB for 7% (68/1,003). Table 1 shows the demographic and epidemiologic characteristics of the cohort.

**Table 1:** Demographic and clinical characteristics of all notified TB cases in Cologne 2012–2022 as well as of the analysed EPTB subgroup 2012–2019

Characteristics		Notified and reported TB cases *N* = 1,*003*			EPTB subgroup analysis *N* = 254			
Sex								
Male		638 (63.6)			142 (55.9)			
Female		360 (35.9)			112 (44.1)			
Divers		2 (0.2)			-			
missing		3 (0.3)			-			
WHO region of origin								
Europe		*These numbers are provided in* Fig. 2.			109 (42.9)			
Africa					53 (20.9)			
South-East Asia					44 (17.3)			
Eastern Mediterranean					44 (17.3)			
Western Pacific					2 (0.8)			
The Americas					2 (0.8)			
TB incidence in country of origin cases per 100,000 population								
< 10		397 (39.6)			73 (28.7)			
10–100		339 (33.8)			74 (29.1)			
> 100		267 (26.6)			107 (42.1)			
Mean age at TB diagnosis, years [± SD]		44.0 [± 20.9]			43.2 [± 20.8]			
born in Germany			50.7 [± 22.7]	*p* < 0.001		54.1 [± 23.1]		*p* < 0.001
foreign born			40.6 [± 19.0]			38.4 [± 17.6]		
TB manifestation								
PTB		674 (67.2)			-			
EPTB		261 (26.0)			199 (78.3)			
EPTB + PTB		68 (6.8)			55 (21.7)			
Mean TB incidence, cases per 100,000 inhabitants		8.1			-			
PTB		5.7			-			
EPTB		2.6			-			
EPTB Affected Organs								
Lymph node		-			152 (50.8)			
Pleura		-			52 (17.4)			
Abdomen/Digestive system		-			30 (10.0)			
Bone/Spinal column		-			29 (9.7)			
Genitourinary tract		-			17 (5.7)			
ENT area		-			8 (2.7)			
CNS		-			4 (1.3)			
Pericard		-			4 (1.3)			
Skin		-			3 (1.0)			
Drugs used for treatment in EPTB								
INH		-			240 (94.5)			
RMP		-			249 (98.0)			
EMB		-			240 (94.5)			
PZA		-			243 (95.7)			
MFX		-			18 (7.1)			
LFX		-			3 (1.2)			
AMK		-			3 (1.2)			
LZD		-			2 (0.8)			
SM		-			1 (0.4)			
CFZ		-			1 (0.4)			
TRD		-			1 (0.4)			
PTO		-			2 (0.8)			
Drug Resistance		83 (8.3)			20 (7.9)			
Mono-resistance		45 (4.5)			13 (5.1)			
Poly-resistance		4 (0.4)			1 (0.4)			
MDR		19 (1.9)			1 (0.4)			
(pre)XDR		-			-			
missing		182 (18.1)			92 (36.2)			
Treatment outcome								
Successful treatment		795 (79.3)			226 (89.0)			
Ongoing treatment		29 (2.9)			3 (1.2)			
Treatment discontinued		21 (2.1)			6 (2.4)			
Treatment failure		1 (0.1)			-			
Death of TB		71 (7.1)			6 (2.4)			
Death of other cause		19 (1.9)			3 (1.2)			
Treatment result unknown		65 (64.8)			10 (3.9)			
missing		2 (0.2)			-			
TB diagnostics					*positive*		*negative*	
PCR	tissue	-			161 (79.3)		42 (20.7)	
	sputum	-			22 (13.8)		138 (86.3)	
Microscopy	tissue	-			118 (52.2)		108 (47.8)	
	sputum	-			8 (3.7)		208 (96.3)	
Culture	tissue	-			163 (82.7)		34 (17.3)	
	sputum	-			45 (26.5)		125 (73.5)	

Number (percentage)

Comparison of means using t-test; statistical significance considered at *p*-values < 0.05, demanding 95% confidence intervals

*TB* tuberculosis, *EPTB* extrapulmonary tuberculosis, *PTB* pulmonary tuberculosis, *WHO* World Health Organisation, *SD* standard deviation, *ENT* ear nose throat, *CNS* central nervous system, *INH* isoniazid, *RMP* rifampicin, *EMB* ethambutol, *PZA* pyrazinamide, *MFX* moxifloxacin, *LFX* levofloxacin, *AMK* amikacin, *LZD* linezolid, *SM* streptomycin, *CFZ* clofazimine, *TRD* terizidone, *PTO* protionamide, *MDR* multi drug resistance, *XDR* extensive drug resistance, *PCR* polymerase chain reaction

**Fig. 2:**
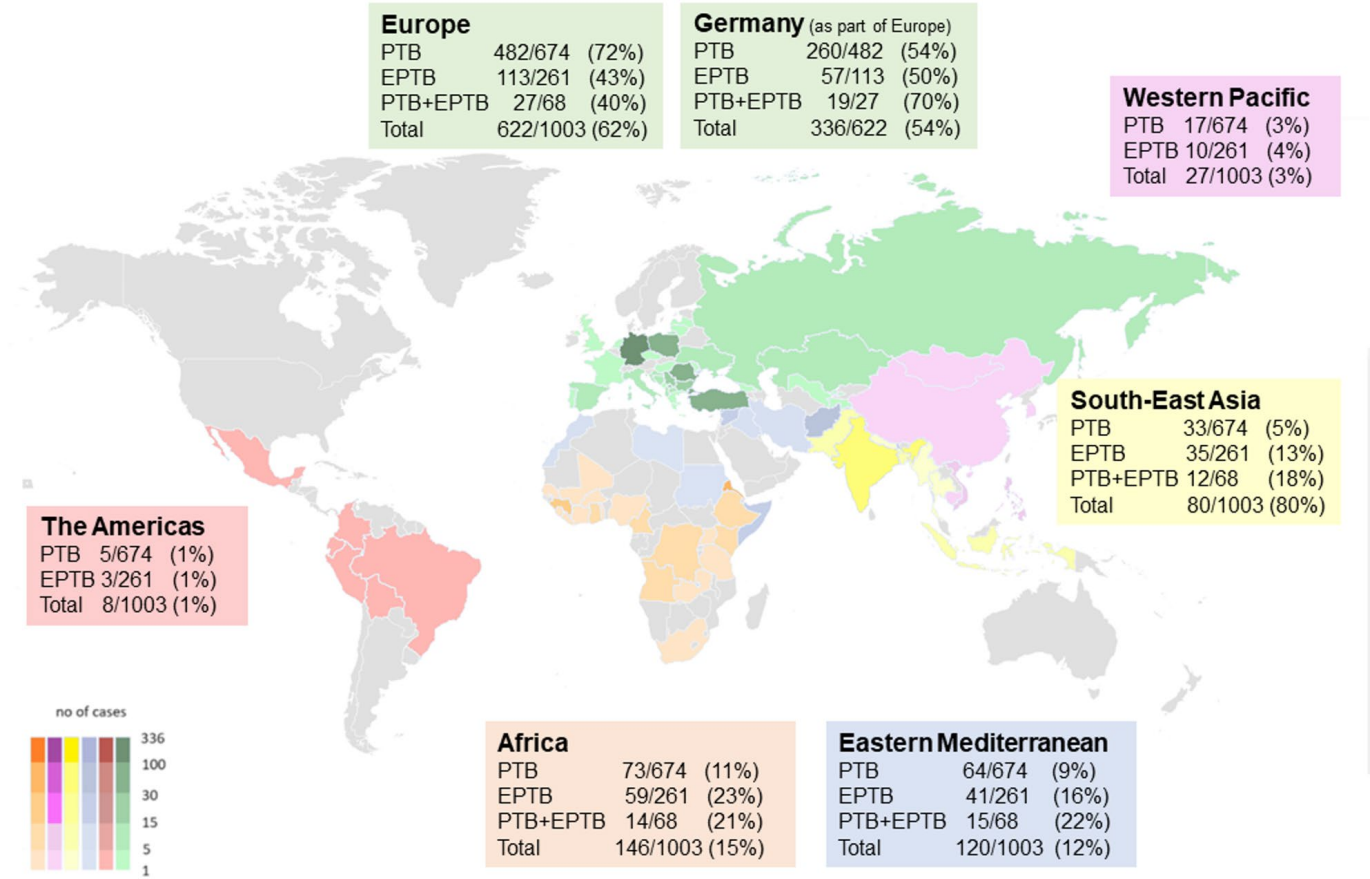
Geographical distribution of the countries of origin of the reported TB cases in Cologne from 2012–2022 (*n* = 1,003) according to the WHO regions. Germany as part of the WHO Europe region is presented separately due to its high contribution to the number of cases. PTB, pulmonary tuberculosis; EPTB, extrapulmonary tuberculosis

Most individuals originated from the WHO European region, followed by the African and Eastern Mediterranean region (Fig. 2).

Notably, a significantly higher proportion of male patients was observed in the Eastern Mediterranean region (*p* = 0.002). The cohort included a total of 164 individuals (16.4%) classified as asylum seekers, with an average age of 27.3 years [SD ± 14.0]. We found no significant difference in pulmonary or extrapulmonary TB manifestation based on residence status (*p* = 0.701).

**Fig. 3:**
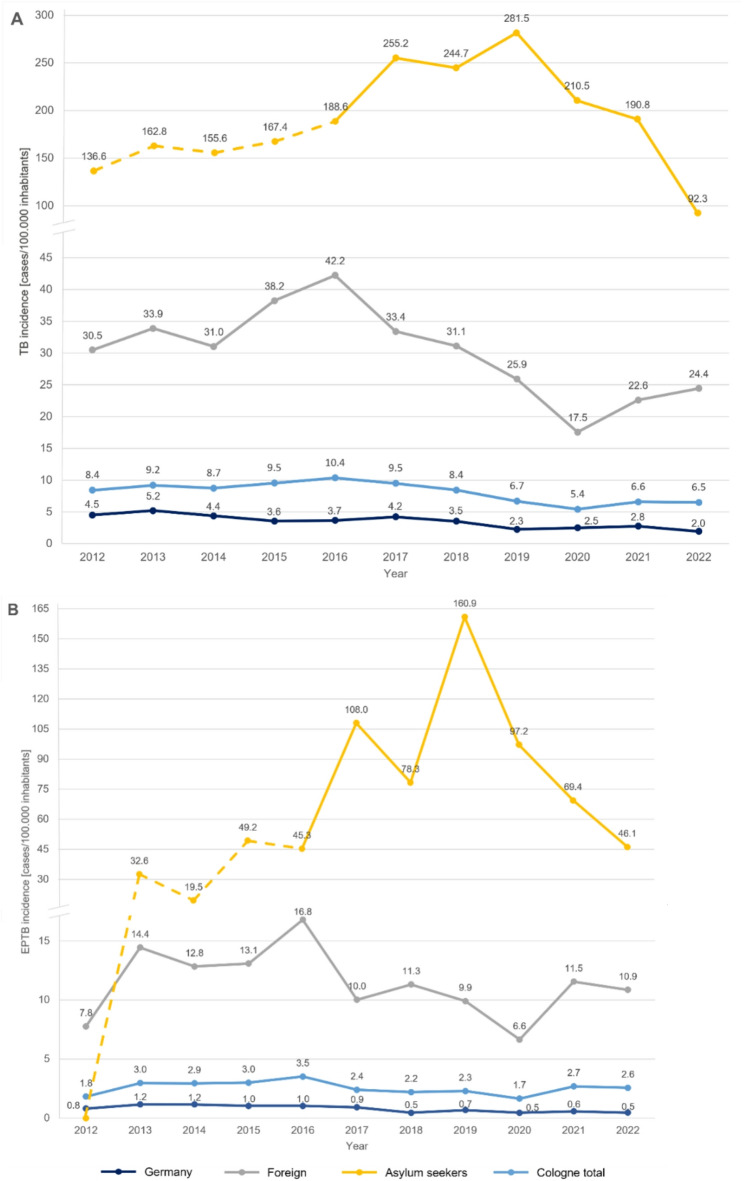
Cologne’s TB (**A**) and EPTB (**B**) incidences over time between 2012–2022 for persons born in Germany, foreign born persons, and for persons with the residence status “asylum seeker”. The line for the latter is only shown as a dashed line for the years 2012–2015, as there were no instructions for documenting the residence status within the authority and the entries could be incomplete. The overall incidence of (EP)TB in Cologne was added for orientation purposes and consists of all reported cases regardless of origin or residence state. TB, tuberculosis; EPTB, extrapulmonary tuberculosis

Cologne’s mean annual incidence of TB during the observation period was 8.1 per 100,000 population. Figure 3 illustrates the incidence of TB and EPTB among different population groups.

**Fig. 4:**
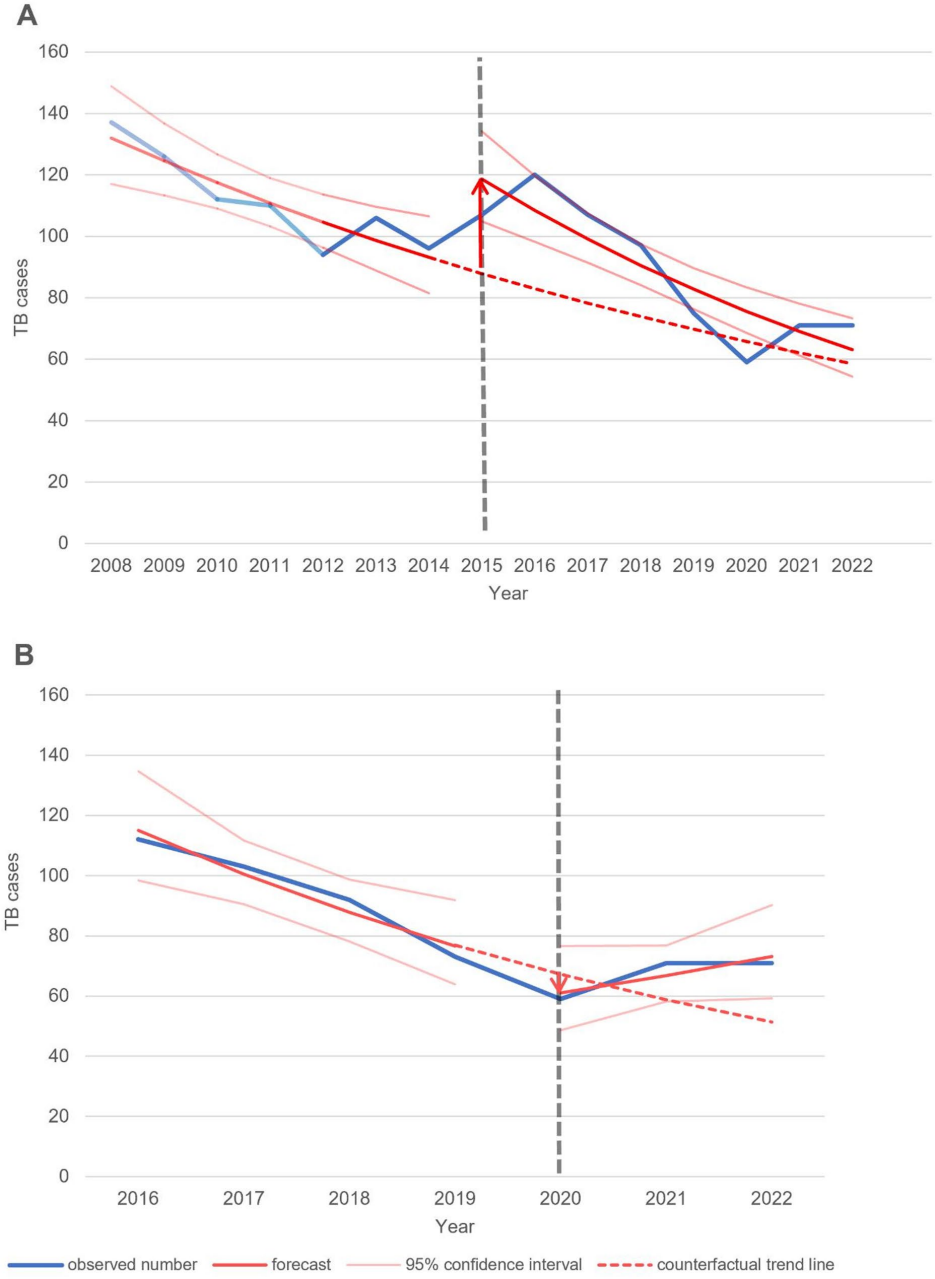
Interrupted time series analysis (ITSA) of the events of the 2015 European migration crisis (**A**) and the start of the SARS-CoV-2 pandemic in Germany in 2020 (**B**). The counterfactual trend line illustrates the course without the event. In subfigure A, the 2008–2011 section is shown in a paler colour, as the regional number of reported TB cases for Cologne used here are taken from Robert Koch-Institute reports [15, 16]. TB, tuberculosis

The highest and lowest calculated incidence values for EPTB in Cologne were observed in 2016 (3.5/100,000 inhabitants) and 2020 (1.7/100,000 inhabitants) respectively (Fig. 3). These years are closely linked to Germany being affected by the European migration crisis (2015) and the start of the SARS-CoV-2 pandemic (2020). The results of the ITSAs carried out for these events showed a significant increased level of the number of TB cases for the European migration crisis (*p* = 0.006), but no significant slope change (*p* = 0.191) with a continuing downward trend (Fig. 4A). For the SARS-CoV-2 pandemic, significant changes in both slope and level were observed (*p* = 0.022): a significant decline of TB cases in 2020 was followed by increasing numbers contrary to the downward trend that had continued until then (Fig. 4B).

Regarding drug resistance, the majority of all TB cases were caused by pan-susceptible pathogens (738/1,003; 74%) (Table 1). Individuals from the European region showed significantly more pan-susceptible strains (*p* = 0.031). Occurrence of multidrug-resistant strains did not differ between EPTB and PTB.

**Fig. 5:**
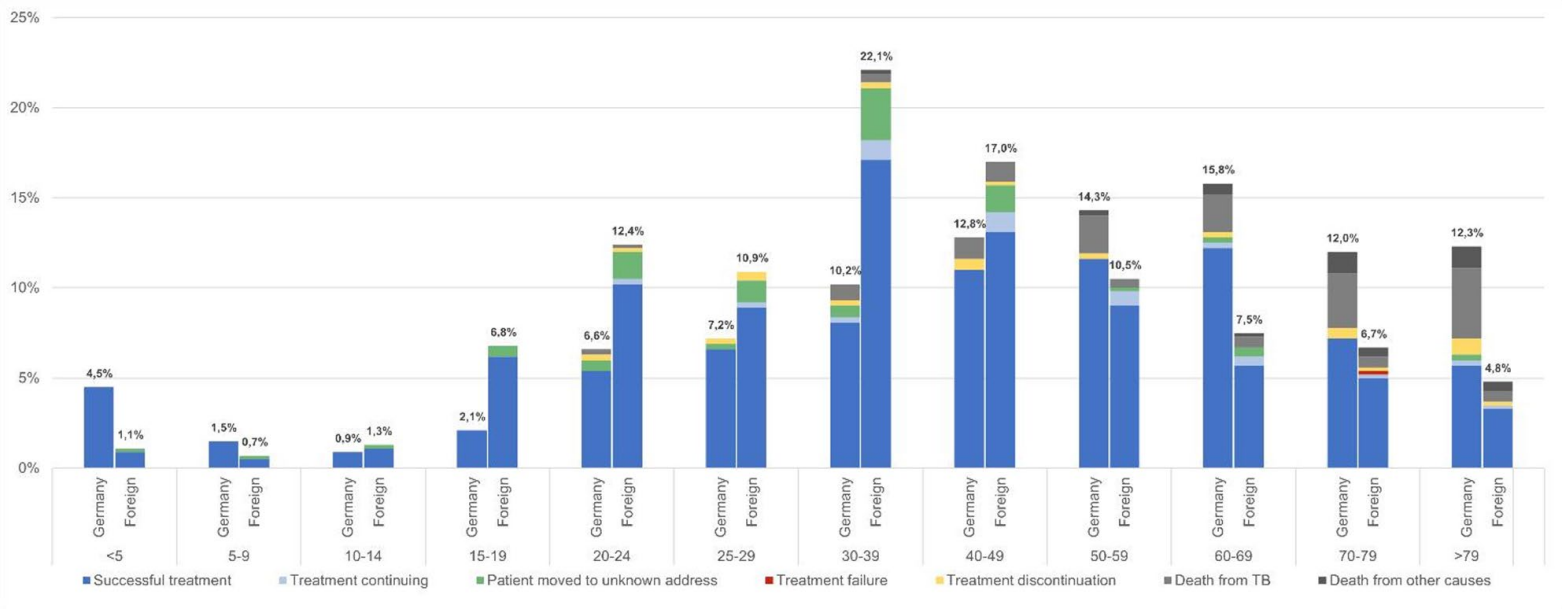
Distribution of outcome categories across age groups depending on the patients’ place of birth. A distinction was made between people born in Germany and those foreign born. The percentages given refer to the respective total number of people with known outcome data born in Germany (*n* = 335) or foreign born (*n* = 666)

Treatment outcomes were available for 1,001 of the 1,003 patients. Figure 5 depicts the distribution across age groups depending on the patients’ place of birth. Successful treatment was overall reported in 79% (Table 1). For PTB, successful treatment outcomes were reported in 78% (523/673) of cases, while for isolated EPTB this result was achieved in 82% (213/260).

The number of TB-related deaths increased progressively from the 30–39 age group onward, reaching a peak in those aged 79 and older. Non-TB-related deaths were predominantly in individuals aged over 60 (17/19, 90%).

There was no significant difference in treatment outcomes by gender. However, we observed a significant correlation between treatment outcome and patients’ country of birth (*p* < 0.001): Patients born in Germany were more likely to die from TB, while treatment outcome could frequently not be determined in foreign born individuals as they had left the health authority’s area of responsibility. This trend also persisted when examining residence status: asylum seekers were significantly more likely to move between areas of jurisdiction compared to regular residents (*p* < 0.001).

### EPTB subgroup analysis

#### Gender and regional patterns

Subgroup analysis of the 254 EPTB patients with detailed report files revealed a significantly higher proportion of women in isolated EPTB cases (91/199, 46%) compared to isolated PTB (167/546, 31%, *p* = 0.001) within the same observation period. The majority of individuals originated from countries with high TB incidence (107/254, 42%), whereas only 19% (106/546) of isolated PTB had this origin (*p* < 0.0001). Information on regional distribution is depicted in Table 1.

#### EPTB diagnosis

Diagnostics for EPTB were mostly triggered by suspicious symptoms (219/246, 89%), followed by incidental findings (14/254, 6%), while other reasons played only a minor role. Symptoms of fever (*p* = 0.014), weight loss (*p* < 0.001), and night sweats (*p* = 0.105) were less frequently observed in patients with isolated EPTB compared to those with additional pulmonary involvement. Patients with EPTB predominantly presented with unexplained swelling of regional lymph nodes. Computed tomography of the chest and/or abdomen (168/254, 66%) and ultrasonography (108/254, 43%) were the most frequently employed diagnostic imaging techniques.

Interferon-gamma release assay (IGRA) was documented in 54% (137/254) of patients, with 118 (86%) yielding positive results. Tissue culture provided the highest rate of direct pathogen detection (163/197, 83%), compared to microscopy or PCR of tissue samples (Table 1).

#### Pathogen identification

*M. tuberculosis* was identified as the causative pathogen of EPTB in 88% (223/254). In 5 cases, other causative mycobacteria were identified, including one case of *M. africanum* and four cases of *M. bovis.* Three of the latter cases involved individuals from the Middle East (Turkey and Syria), and one from Ethiopia. Out of the reported EPTB cases, 26 (10%) had no documented pathogen.

#### Affected organs

Lymph node involvement was most common (Table 1). Disseminated disease, defined as infection involving 2 or more non-contiguous sites, was found in 11% (21/199) of isolated EPTB. When cases with lung involvement were added, the number increased to 30% (76/254).

#### Comorbidity

Haematological and oncological diagnoses were particularly common within EPTB subgroup (70/254, 28.1%). The majority were anaemias, with microcytic anaemia being the most common subtype (32/70, 45.7%), attributable to either iron deficiency or haemoglobinopathies.

Another important factor was diabetes mellitus with 23 cases (9.2%). HIV co-infection was documented in 11 people (4.3%). Overall, a health condition associated with immunosuppression was present in 99 cases (39.3%). No cases of drug-induced systemic immunosuppression were reported.

#### Treatment in EPTB

The median duration of therapy was 210 days (IQR: 180–300) with a median duration of the initial phase of 60 days (IQR: 60–75). The continuation phase had a median duration of 131 days (IQR: 120–210). The average treatment duration for cases involving bone and joint manifestations was 12.7 months, while for affection of the central nervous system (CNS), it was 12.5 months. The initial phases lasted on average 2.6 and 2.3 months, the continuation phases 10.1 and 10.3 months.

The mean duration of inpatient treatment for isolated EPTB was 14.5 days (SD ± 13.7). A hospitalisation of less than 3 weeks was significantly more frequent in isolated EPTB (*p* = 0.048).

With regard to the drug treatment regimes, the standard therapy was administered in 90.6% (230/254). Fluoroquinolones were utilised in instances of confirmed resistance to standard drugs or as replacements due to adverse effects (Table 1).

#### Drug-related adverse effects

Drug-related adverse effects occurred in 34% (84/247; 7 missings). Drug-induced liver injury (DILI) was the most common (10%), followed by allergic reactions (4%). Visual impairment occurred only 4 times (2%). A correlation with the administration of EMB could not be statistically proven (*p* = 1.000). However, the occurrence of adverse effects was statistically significantly more frequently correlated with the administration of EMB (*p* = 0.018) or fluoroquinolones (moxifloxacin *p* = 0.004; levofloxacin *p* = 0.038).

While existence of immunosuppression and the general occurrence of side effects correlated (*p* = 0.006), no correlation was observed regarding gender (*p* = 0.601) or EPTB affected organs.

## Discussion

Our study sought to provide a comprehensive overview of EPTB incidence in an urban area of a low-incidence country over a recent ten-year period. EPTB accounted for 26% of TB patients, with lymph node (51%) and pleural TB (17%) being the most common forms, consistent with national and international trends [5, 7, 17]. In line with prior research, we observed a significantly higher proportion of isolated EPTB among females compared to PTB, despite a male predominance in absolute numbers for both groups [18, 19]. This gender disparity in TB presentation warrants further investigation to elucidate underlying factors, including as biological, social, and healthcare-seeking behaviours [20–22].

Our study found higher TB incidences in Cologne compared to national references provided by the RKI [5]. This aligns with the recognised trends showing that urban areas have higher concentrations of vulnerable groups, including homeless individuals, drug users, and migrants from high-incidence countries [23–25]. The allocation of approximately 10,000 asylum seekers to Cologne during the European migration crisis of 2015–2016 coincides with our study period [26]. We demonstrated through ITSA that this migration movement significantly impacted (*p* = 0.006) the incidence of TB in Cologne as it raised the number of notified TB cases to another level. A sustained change in the trend could not be observed, which suggests that the 2015 crisis was a narrowly circumscribed event and that the previously observed trend in the general population continued after the detection of the immigrant TB cases. A substantial proportion of these immigrants originated from regions with higher TB incidences such as Syria and Iraq [10, 27], aligning with the sizable representation from the WHO Eastern Mediterranean region in our cohort. Asylum seekers, predominantly male and younger than German-born TB patients (average age 27.3 years vs. 50.7 years), exhibited a TB incidence up to 100 times higher than German-born individuals.

TB was also more frequent among individuals from Eastern European countries like Poland, Bulgaria, and Romania [10], with some of these countries historically reporting significantly higher TB incidences compared to Germany [28]. This is particularly relevant given the ongoing conflict between Ukraine and Russia leading to migration from Eastern to Western Europe. Initial reports indicate an increase in TB cases and changes in TB incidence in Germany and neighbouring countries due to refugees from Ukraine [5, 29–31], which counteract the goal of reducing TB incidence by 50% by 2025 in accordance with the Tuberculosis action plan for the WHO European Region, 2023–2030 [32]. The significant role of migration in TB incidence is further evidenced by the notable impact (*p* = 0.022) of the onset of the SARS-CoV-2 pandemic observed in the ITSA for Cologne. However, it is essential to note that alongside the nearly complete halt of migration movements due to the lockdown measures, underreporting is also a likely factor attributed to limited medical access and constrained official surveillance capacities [33]. The observed change in the trend contrary to the assumed further downward trend may be attributable to delayed diagnoses that only occurred after the lockdown measures had ended. Therefore, further exploration should focus on the implications of migration on TB control efforts, healthcare access for migrants, and the need for targeted interventions to address TB among migrant populations.

Although PTB incidence among asylum seekers had stabilised by 2017, we observed a rise in EPTB incidence until 2019. Active case finding by German health authorities may partially explain this finding. Notably, active case finding in Germany prioritises PTB diagnosis [34, 35], potentially delaying EPTB recognition due to its nonspecific symptoms. Our cohort revealed that EPTB often lacked the typical B symptoms, i.e. unintentional weight loss, night sweats and/or fever, associated with TB. Instead, it presented with unexplained lymph node swelling, which may lead to consideration of alternative diagnoses more common in Western countries. Greater awareness among healthcare providers, enhanced diagnostic tools, and early detection strategies are critical to improving EPTB outcomes.

In about one-third of EPTB cases, drug-related side effects were reported, with drug-induced liver injury (DILI) being the most common. While DILI is well-documented with RMP, INH, and PZA [36, 37], no correlation to a specific drug was found. In our cohort, fluoroquinolones appeared to present a higher side-effect potential. Overall, standard therapies were well tolerated, as indicated by the low rate of discontinuation. In Germany, treatment success depends to a large extent on patient self-management and the physician’s adherence to standardised treatment protocols. For instance, the national guidelines recommend a six-month duration for lymph node TB and a twelve-month durations for CNS or disseminated TB [12]. In this context, we observed that treatment durations generally aligned with these guidelines, implying both patient compliance and physician guideline adherence. This is likely due to the concentration of EPTB treatment in specialised centres and may contribute to the slightly better treatment success rate of EPTB (82%) we noticed in comparison to PTB (78%).

However, deviations from the standard regimen were observed in approximately 9.4% of cases, where fluoroquinolones or other second-line agents were administered due to confirmed resistance or clinical decisions tailored to patient-specific conditions.

The limited data available on treatment adherence and duration in low-incidence countries, particularly for EPTB, underscores the significance of this analysis. Most existing studies focus on PTB or derive data from high-incidence settings, where directly observed therapy (DOT) or other treatment supporting strategies are more commonly employed. In contrast, our findings suggest that EPTB care in low-incidence settings could benefit from the development of more robust follow-up strategies and adherence monitoring protocols. Our observed cure rate (82%) falls below the WHO´s target of 90% [1]. The reasons for this discrepancy remain unclear but may be multifactorial, involving factors such as ambiguities in EPTB definitions, reporting challenges, and methodological issues like loss to follow-up. Furthermore, TB affects population groups like refugees who may not necessarily access health and welfare system.

Addressing these issues could also contribute to improved treatment success rates and better alignment with WHO targets.

### Limitations

Our study has several limitations. Firstly, our analysis relied mostly on data available to the health authority in Cologne, which may have resulted in incomplete information if monitored individuals moved out of the authority’s jurisdiction. The transient nature of migration, as individuals may continue to migrate even after arriving in their destination country, highlights the importance of consistent documentation and transfer of treatment records. Secondly, inconsistencies in residence status data prior to 2016 are possible. The numbers reported for asylum seekers prior to 2016 represent a minimum estimate. Additional individuals may have held this residence status, meaning that the observed differences compared with regular residents were likely underestimated. Reporting delays, especially around year-end, could also contribute to observed differences compared to national RKI statistics.

Our study period represents a period of historically low TB incidence in Germany, while at the same time coinciding with a phase of active case finding during the peak of the refugees’ arrivals which may have influenced the observed trends [38].

## Conclusion

EPTB represents a public health concern, potentially causing substantial chronic disability and loss in quality of life, particularly among migrant and refugee populations facing healthcare barriers. EPTB’s nonspecific symptoms and delayed diagnoses highlight the need for enhanced and targeted diagnostic and treatment strategies in low-incidence countries. Collaborative efforts among clinicians, public health officials, and policymakers as demanded by the Tuberculosis action plan for the WHO European Region, 2023–2030 [32], are crucial to achieving WHO End TB Strategy goals, especially for neglected forms of TB like EPTB.

## Data Availability

The dataset analysed in the current study is available from the corresponding author on reasonable request.

## Abbreviations

CI: Confidence interval
CNS: Central nervous system
COVID-19: Coronavirus disease 2019
DILI: Drug induced liver injury
DOT: Directly observed therapy
EMB: Ethambutol
EPTB: Extrapulmonary tuberculosis
HIV: Human immunodeficiency virus
INH: Isoniazid
IQR: Interquartile range
ITSA: Interrupted time series analysis
MDR: Multi drug resistance
PCR: Polymerase chain reaction
PTB: Pulmonary tuberculosis
PZA: Pyrazinamid
RKI: Robert Koch-Institut
RMP: Rifampicin
SARS-CoV-2: Severe acute respiratory syndrome coronavirus type 2
SD: Standard deviation
TB: Tuberculosis
WHO: World Health Organization
XDR: Extensive drug resistance
